# (*E*)-1-Methyl-4-[2-(1-naphth­yl)vin­yl]pyridinium 4-bromo­benzene­sulfonate^1^

**DOI:** 10.1107/S1600536809014974

**Published:** 2009-04-30

**Authors:** Suchada Chantrapromma, Kullapa Chanawanno, Hoong-Kun Fun

**Affiliations:** a Crystal Materials Research Unit, Department of Chemistry, Faculty of Science, Prince of Songkla University, Hat-Yai, Songkhla 90112, Thailand; b X-ray Crystallography Unit, School of Physics, Universiti Sains Malaysia, 11800 USM, Penang, Malaysia

## Abstract

In the title compound, C_18_H_16_N^+^·C_6_H_4_BrO_3_S^−^, the cation exists in the *E* configuration and the whole mol­ecule of the cation is disordered with a refined site-occupancy ratio of 0.733 (1):0.267 (1). The naphthalene system is not planar, the inter­planar angle between the two aromatic rings being 5.0 (5)° for the major component and 5.7 (10)° for the minor component. The cation is twisted with dihedral angles between the pyridinium ring and the two aromatic rings of the naphthalene system of 56.3 (5) and 51.4 (5)° (for the major component) and 52.2 (11) and 53.4 (11)° (for the minor component). The pyridinium ring and the benzene ring of the anion are inclined to each other at inter­planar angles of 85.0 (4) and 71.5 (9)° for the major and minor components, respectively. In the crystal packing, the cations and anions are alternately arranged with the cations stacked in an anti­parallel manner along the *c* axis and the anions linked together into chains along the same direction. The cations are linked to the anions into chains along [102] by weak C—H⋯O inter­actions. The crystal structure is further stabilized by C—H⋯π inter­actions and π–π contacts, with *Cg*⋯*Cg* distances of 3.502 (9) and 3.698 (6) Å. A short Br⋯O contact [3.029 (4) Å] is also present.

## Related literature

For bond-length data, see: Allen *et al.* (1987 ▶). For background to NLO materials research, see: Cheng *et al.* (1991*a* ▶; 1991*b* ▶); Dittrich *et al.* (2003 ▶); Ogawa *et al.* (2008 ▶); Weir *et al.* (2003 ▶); Yang *et al.* (2007 ▶). For related structures, see, Chanawanno *et al.* (2008 ▶) and Chantrapromma *et al.* (2006 ▶; 2007 ▶; 2008 ▶; 2009 ▶). For the stability of the temperature controller used in the data collection, see Cosier & Glazer, (1986 ▶).
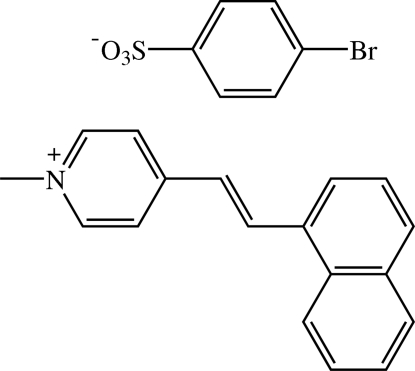


## Experimental

### 

#### Crystal data

C_18_H_16_N^+^·C_6_H_4_BrO_3_S^−^
*M* *_r_* = 482.38
Orthorhombic, 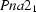

*a* = 12.2195 (2) Å
*b* = 21.9907 (4) Å
*c* = 7.6256 (1) Å
*V* = 2049.12 (6) Å^3^
*Z* = 4
Mo *K*α radiation
μ = 2.14 mm^−1^
*T* = 100 K
0.46 × 0.15 × 0.14 mm

#### Data collection

Bruker APEXII CCD area-detector diffractometer
Absorption correction: multi-scan (*SADABS*; Bruker, 2005 ▶) *T*_min_ = 0.437, *T*_max_ = 0.753
15171 measured reflections
5563 independent reflections
3975 reflections with *I* > 2σ(*I*)
*R*_int_ = 0.044

#### Refinement

*R*[*F* ^2^ > 2σ(*F* ^2^)] = 0.047
*wR*(*F* ^2^) = 0.107
*S* = 1.03
5563 reflections
326 parameters
11 restraints
H-atom parameters constrained
Δρ_max_ = 1.11 e Å^−3^
Δρ_min_ = −0.49 e Å^−3^
Absolute structure: Flack (1983 ▶), 2373 Friedel pairs
Flack parameter: −0.003 (11)

### 

Data collection: *APEX2* (Bruker, 2005 ▶); cell refinement: *SAINT* (Bruker, 2005 ▶); data reduction: *SAINT*; program(s) used to solve structure: *SHELXTL* (Sheldrick, 2008 ▶); program(s) used to refine structure: *SHELXTL*; molecular graphics: *SHELXTL*; software used to prepare material for publication: *SHELXTL* and *PLATON* (Spek, 2009 ▶).

## Supplementary Material

Crystal structure: contains datablocks global, I. DOI: 10.1107/S1600536809014974/sj2603sup1.cif

Structure factors: contains datablocks I. DOI: 10.1107/S1600536809014974/sj2603Isup2.hkl

Additional supplementary materials:  crystallographic information; 3D view; checkCIF report

## Figures and Tables

**Table 1:** Hydrogen-bond geometry (Å, °)

*D*—H⋯*A*	*D*—H	H⋯*A*	*D*⋯*A*	*D*—H⋯*A*
C2*A*—H2*AA*⋯O1^i^	0.95	2.53	3.392 (11)	151
C5*A*—H5*AA*⋯O2^ii^	0.95	2.47	3.362 (10)	157
C11*A*—H11*A*⋯O1^i^	0.95	2.34	3.282 (10)	175
C14*A*—H14*A*⋯O2^iii^	0.95	2.44	3.373 (7)	169
C16*A*—H16*A*⋯O3^iv^	0.95	2.49	3.361 (11)	153
C17*A*—H17*A*⋯O1^i^	0.95	2.35	3.227 (14)	153
C18*A*—H18*A*⋯O3^iv^	0.98	2.57	3.501 (8)	159
C19—H19*A*⋯O2	0.95	2.56	2.930 (5)	103
C20—H20*A*⋯O1^iv^	0.95	2.34	3.252 (5)	161
C22—H22*A*⋯O3^v^	0.95	2.51	3.274 (6)	137
C4*A*—H4*AA*⋯*Cg*2^vi^	0.95	2.84	3.659 (14)	145
C7*A*—H7*AA*⋯*Cg*4^vii^	0.95	2.88	3.657 (9)	140
C4*B*—H4*BA*⋯*Cg*2^vi^	0.95	2.90	3.59 (2)	130

Symmetry codes: (i) 
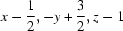
; (ii) 
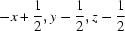
; (iii) 
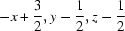
; (iv) 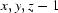; (v) 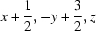; (vi) 
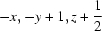
; (vii) 
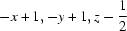
. *Cg*2 and *Cg*4 are the centroids of the C1*A*–C6*A* and C19–C24 rings, respectively.

**Fig. 1.**
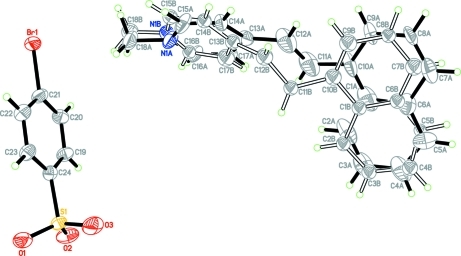
The molecular structure of the title compound, with 50% probability displacement ellipsoids and the atom-numbering scheme. Open bonds show the minor component.

**Fig. 2.**
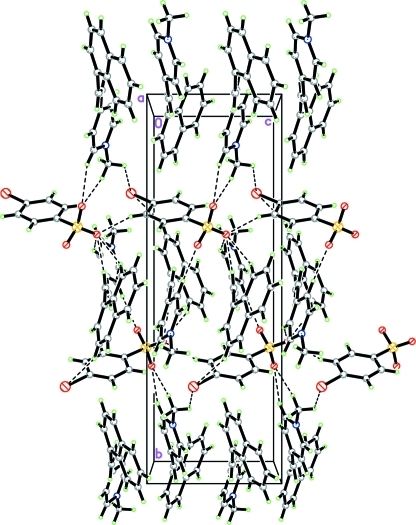
The crystal packing of the major component of the title compound viewed down the a axis. Weak C—H···O interactions are shown as dashed lines.

## supplementary crystallographic information

### Comment

Nonlinear optics has been recognized for several decades as a promising field with important applications in the domain of opto-electronics and photonics. Hence, a variety of materials have been investigated for their nonlinear optical (NLO) properties. In order to obtain second-order NLO single crystals, the main requirements should be the choice of molecules with large hyperpolarizability (*β*) and the alignment of these molecules with optimal orientation into a noncentrosymmetric space group in the crystal. Organic crystals with extensive conjugated π systems with large hyperpolarizability which exhibit NLO properties have been reported (Dittrich *et al.*, 2003; Ogawa *et al.*, 2008; Weir *et al.*, 2004; Yang *et al.*, 2007). Styryl pyridinium derivatives are considered to be good conjugated π-systems (Cheng *et al.*, 1991*a*, 1991*b*). We have previously synthesized and reported the crystal structures of several  pyridinium salts (Chanawanno *et al.*, 2008; Chantrapromma *et al.*, 2006, 2007, 2008, 2009) in order to study their NLO properties. The title compound (I) was synthesized by introducing a naphthalenyl group into the cation in order to increase the extent of π-conjugation in the system. The title compound crystallizes in the orthorhombic non-centrosymmetric space group *P*na2_1_ therefore it should exhibit second-order nonlinear optical properties.

Fig. 1 shows the asymmetric unit of (I) which consists of a C_18_H_16_N^+^ cation and  a C_6_H_4_BrO_3_S^-^ anion. The whole molecule of the cation is disordered over two sites; the major component *A* and the minor component *B* (Fig. 1 ), with the refined site-occupancy ratio of 0.733 (1)/0.267 (1). The cation exists in the *E* configuration with respect to the C11═C12 double bond. The naphthalenyl moiety is not planar as indicated by the interplanar angle between the two aromatic C1–C6 and C1/C6–C10 rings being 5.0 (5)° (for the major component *A*) and 5.7 (10)° (for the minor component *B*). The cation is twisted with the dihedral angle between the pyridinium and the two aromatic C1–C6 and C1/C6–C10 rings being 56.3 (5)° and 51.4 (5)°, respectively (for the major component *A*); 52.2 (11)° and 53.4 (11)°, respectively (for the minor component *B*) and the torsion angles C19–C10–C11–C12 = -26.5 (14)° and C11–C12–C13–C17 = -9.3 (15)° for the major component *A*; whereas the corresponding values are -6(2)° and 4(3)° for the minor component *B*. The cation and anion are inclined to each other with interplanar angles of 85.0 (4)° and 71.5 (9)° respectively between the benzene ring and the pyridinium units of the major and minor disorder components. The bond lengths in (I) are in normal ranges (Allen *et al.*, 1987) and comparable to those in related structures (Chanawanno *et al.*, 2008; Chantrapromma *et al.*, 2006, 2007, 2008, 2009).

In the crystal packing (Fig. 2), all O atoms of the sulfonate group are involved in weak C—H···O interactions (Table 1). The cations and anions are alternately arranged with the cations (both the major *A* and minor *B* components) stacked in an antiparallel manner along the *c* axis and the anions linked together into chains along the same direction. The cations are linked to the anions into chains along the [1 0 2] direction by weak C—H···O interactions (Table 1). The crystal structure is further stabilized by C—H···π interactions (Table 1). π–π interaction with the distances Cg_1_···Cg_2_ = 3.698 (6) Å and Cg_1_···Cg_3_ = 3.502 (9) Å are also observed (symmetry code for both Cg···Cg interactions: 1-x, 1-y,-1/2+z); Cg_1_, Cg_2_, Cg_3_ and Cg_4_ are the centroids of the N1A/C13A–C17A, C1A–C6A, C1B–C6B and C19–C24 rings, respectively.  A short Br1···O3 [3.029 (4) Å] contact is also present.

### Experimental

(*E*)-1-methyl-4-(2-(naphthalen-1-yl)vinyl)pyridinium iodide (compound A) was prepared by mixing solutions of 1,4-dimethylpyridinium iodide (2 g, 8.5 mmol), 1-naphthaldehyde (1.16 ml, 8.5 mmol) and piperidine 0.84 ml, 8.5 mmol) in methanol (40 ml). The resulting solution was refluxed for 3 h under a nitrogen atmosphere. The solid which formed was filtered and washed with chloroform. After purification, the yellow solid of compound A (0.22 g, 0.58 mmol) was mixed with silver 4-bromobenzenesulfonate (Chantrapromma *et al.*, 2006) (0.20 g, 0.58 mmol) in methanol (100 ml) and stirred for 0.5 h. The precipitate of silver iodide was filtered and the filtrate was evaporated to give the title compound as a yellow solid. Yellow needle-shaped single crystals of the title compound suitable for *x*-ray structure determination were recrystallized from methanol by slow evaporation at room temperature over a few weeks, Mp. 495-496 K.

### Refinement

All H atoms were positioned geometrically and allowed to ride on their parent atoms, with d(C-H) = 0.95 Å for aromatic and CH and 0.98 Å for CH_3_ atoms. The *U*_iso_ values were constrained to be 1.5*U*_eq_ of the carrier atom for methyl H atoms and 1.2*U*_eq_ for the remaining H atoms. A rotating group model was used for the methyl groups. The highest residual electron density peak is located at 0.90 Å from C11A and the deepest hole is located at 0.32 Å from C11A. The cation is disordered over two sites with occupancies 0.733 (1) and 0.267 (1) respectively. All atoms of the minor component *B* were refined isotropically. Initially rigid, similarity restraints were applied to the minor component *B*. After a steady state was reached, these restraints were removed before the final refinement.

### Crystal data

**Table:**

C_18_H_16_N^+^·C_6_H_4_BrO_3_S^−^	*D*_x_ = 1.564 Mg m^−^^3^
*M**_r_* = 482.38	Melting point = 495–496 K
Orthorhombic, *P**n**a*2_1_	Mo *K*α radiation, λ = 0.71073 Å
Hall symbol: P 2c -2n	Cell parameters from 5563 reflections
*a* = 12.2195 (2) Å	θ = 2.5–30.0°
*b* = 21.9907 (4) Å	µ = 2.13 mm^−^^1^
*c* = 7.6256 (1) Å	*T* = 100 K
*V* = 2049.12 (6) Å^3^	Needle, yellow
*Z* = 4	0.46 × 0.15 × 0.14 mm
*F*(000) = 984	

### Data collection

**Table:**

Bruker APEXII CCD area-detector diffractometer	5563 independent reflections
Radiation source: sealed tube	3975 reflections with *I* > 2σ(*I*)
graphite	*R*_int_ = 0.044
φ and ω scans	θ_max_ = 30.0°, θ_min_ = 2.5°
Absorption correction: multi-scan (*SADABS*; Bruker, 2005)	*h* = −12→17
*T*_min_ = 0.437, *T*_max_ = 0.753	*k* = −30→25
15171 measured reflections	*l* = −10→10

### Refinement

**Table:**

Refinement on *F*^2^	Secondary atom site location: difference Fourier map
Least-squares matrix: full	Hydrogen site location: inferred from neighbouring sites
*R*[*F*^2^ > 2σ(*F*^2^)] = 0.047	H-atom parameters constrained
*wR*(*F*^2^) = 0.107	*w* = 1/[σ^2^(*F*_o_^2^) + (0.0268*P*)^2^ + 1.3478*P*] where *P* = (*F*_o_^2^ + 2*F*_c_^2^)/3
*S* = 1.03	(Δ/σ)_max_ = 0.002
5563 reflections	Δρ_max_ = 1.11 e Å^−^^3^
326 parameters	Δρ_min_ = −0.48 e Å^−^^3^
11 restraints	Absolute structure: Flack (1983), 2373 Friedel pairs
Primary atom site location: structure-invariant direct methods	Flack parameter: −0.003 (11)

### Special details

**Table:**

Experimental. The crystal was placed in the cold stream of an Oxford Cryosystems Cobra open-flow nitrogen cryostat (Cosier & Glazer, 1986) operating at 100.0 (1) K.
Geometry. All esds (except the esd in the dihedral angle between two l.s. planes) are estimated using the full covariance matrix. The cell esds are taken into account individually in the estimation of esds in distances, angles and torsion angles; correlations between esds in cell parameters are only used when they are defined by crystal symmetry. An approximate (isotropic) treatment of cell esds is used for estimating esds involving l.s. planes.
Refinement. Refinement of F^2^ against ALL reflections. The weighted R-factor wR and goodness of fit S are based on F^2^, conventional R-factors R are based on F, with F set to zero for negative F^2^. The threshold expression of F^2^ > 2sigma(F^2^) is used only for calculating R-factors(gt) etc. and is not relevant to the choice of reflections for refinement. R-factors based on F^2^ are statistically about twice as large as those based on F, and R- factors based on ALL data will be even larger.

### Fractional atomic coordinates and isotropic or equivalent isotropic displacement parameters (Å^2^)

**Table:**

	*x*	*y*	*z*	*U*_iso_*/*U*_eq_	Occ. (<1)
Br1	1.09367 (3)	0.735910 (17)	0.33953 (7)	0.03638 (11)	
S1	0.77513 (7)	0.83524 (5)	0.94982 (13)	0.0282 (2)	
O1	0.8448 (2)	0.85394 (18)	1.0921 (4)	0.0511 (9)	
O2	0.7122 (2)	0.88360 (13)	0.8744 (4)	0.0441 (8)	
O3	0.7095 (3)	0.78212 (14)	0.9922 (5)	0.0518 (9)	
N1A	0.7710 (6)	0.6185 (4)	0.1666 (13)	0.0408 (19)	0.708 (6)
C1A	0.2235 (8)	0.4733 (4)	0.2313 (16)	0.0658 (14)	0.708 (6)
C2A	0.2047 (9)	0.5344 (4)	0.3012 (16)	0.061 (3)	0.708 (6)
H2AA	0.2583	0.5650	0.2827	0.073*	0.708 (6)
C3A	0.1123 (9)	0.5484 (5)	0.3922 (16)	0.0658 (14)	0.708 (6)
H3AA	0.1022	0.5883	0.4370	0.079*	0.708 (6)
C4A	0.0335 (10)	0.5048 (4)	0.4194 (18)	0.0658 (14)	0.708 (6)
H4AA	−0.0282	0.5156	0.4885	0.079*	0.708 (6)
C5A	0.0378 (8)	0.4470 (5)	0.3538 (16)	0.054 (3)	0.708 (6)
H5AA	−0.0208	0.4190	0.3685	0.065*	0.708 (6)
C6A	0.1369 (10)	0.4309 (5)	0.2605 (17)	0.047 (3)	0.708 (6)
C7A	0.1552 (9)	0.3698 (4)	0.1942 (13)	0.057 (3)	0.708 (6)
H7AA	0.0989	0.3401	0.2024	0.069*	0.708 (6)
C8A	0.2557 (7)	0.3547 (3)	0.1185 (10)	0.0410 (18)	0.708 (6)
H8AA	0.2682	0.3142	0.0800	0.049*	0.708 (6)
C9A	0.3383 (7)	0.3982 (3)	0.0984 (10)	0.0401 (18)	0.708 (6)
H9AA	0.4057	0.3868	0.0459	0.048*	0.708 (6)
C10A	0.3236 (5)	0.4569 (3)	0.1531 (12)	0.055 (2)	0.708 (6)
C11A	0.4093 (6)	0.5027 (4)	0.1523 (13)	0.0658 (14)	0.708 (6)
H11A	0.3894	0.5443	0.1429	0.079*	0.708 (6)
C12A	0.5166 (7)	0.4881 (3)	0.1644 (12)	0.0658 (14)	0.708 (6)
H12A	0.5361	0.4470	0.1858	0.079*	0.708 (6)
C13A	0.6044 (5)	0.5338 (3)	0.1455 (9)	0.0354 (15)	0.708 (6)
C14A	0.7055 (6)	0.5194 (3)	0.2165 (8)	0.0304 (14)	0.708 (6)
H14A	0.7179	0.4795	0.2597	0.036*	0.708 (6)
C15A	0.7866 (6)	0.5609 (4)	0.2254 (11)	0.0426 (19)	0.708 (6)
H15A	0.8555	0.5497	0.2734	0.051*	0.708 (6)
C16A	0.6701 (8)	0.6347 (5)	0.0899 (16)	0.032 (2)	0.708 (6)
H16A	0.6591	0.6737	0.0394	0.038*	0.708 (6)
C17A	0.5912 (11)	0.5933 (6)	0.091 (2)	0.034 (2)	0.708 (6)
H17A	0.5207	0.6053	0.0517	0.041*	0.708 (6)
C18A	0.8611 (5)	0.6636 (3)	0.1790 (13)	0.049 (2)	0.708 (6)
H18A	0.8357	0.7030	0.1345	0.074*	0.708 (6)
H18B	0.9235	0.6497	0.1089	0.074*	0.708 (6)
H18C	0.8835	0.6679	0.3017	0.074*	0.708 (6)
N1B	0.7857 (17)	0.6237 (10)	0.118 (3)	0.028 (5)*	0.292 (6)
C1B	0.2380 (11)	0.4829 (7)	0.2936 (15)	0.028 (4)*	0.292 (6)
C2B	0.2071 (16)	0.5389 (8)	0.3799 (19)	0.027 (4)*	0.292 (6)
H2BA	0.2576	0.5711	0.3981	0.032*	0.292 (6)
C3B	0.0975 (14)	0.5426 (9)	0.435 (2)	0.029 (4)*	0.292 (6)
H3BA	0.0726	0.5797	0.4849	0.035*	0.292 (6)
C4B	0.0193 (17)	0.4919 (8)	0.420 (3)	0.032 (4)*	0.292 (6)
H4BA	−0.0524	0.4921	0.4682	0.038*	0.292 (6)
C5B	0.0648 (15)	0.4427 (10)	0.324 (3)	0.020 (4)*	0.292 (6)
H5BA	0.0163	0.4100	0.2996	0.024*	0.292 (6)
C6B	0.1667 (19)	0.4362 (14)	0.264 (4)	0.028 (6)*	0.292 (6)
C7B	0.1927 (16)	0.3832 (10)	0.183 (3)	0.037 (5)*	0.292 (6)
H7BA	0.1410	0.3514	0.1690	0.044*	0.292 (6)
C8B	0.3015 (16)	0.3782 (11)	0.119 (2)	0.025 (4)*	0.292 (6)
H8BA	0.3218	0.3447	0.0472	0.030*	0.292 (6)
C9B	0.3804 (14)	0.4237 (8)	0.164 (2)	0.038 (4)*	0.292 (6)
H9BA	0.4533	0.4190	0.1223	0.045*	0.292 (6)
C10B	0.3572 (12)	0.4713 (7)	0.258 (2)	0.034 (4)*	0.292 (6)
C11B	0.4442 (9)	0.5144 (5)	0.345 (2)	0.027 (3)*	0.292 (6)
H11B	0.4222	0.5506	0.4033	0.032*	0.292 (6)
C12B	0.5668 (9)	0.4966 (6)	0.334 (3)	0.035 (3)*	0.292 (6)
H12B	0.5946	0.4622	0.3948	0.042*	0.292 (6)
C13B	0.6410 (16)	0.5363 (7)	0.221 (2)	0.027 (3)*	0.292 (6)
C14B	0.7498 (16)	0.5288 (9)	0.241 (2)	0.025 (4)*	0.292 (6)
H14B	0.7751	0.4910	0.2865	0.030*	0.292 (6)
C15B	0.8236 (15)	0.5706 (8)	0.202 (2)	0.028 (4)*	0.292 (6)
H15B	0.8988	0.5652	0.2299	0.034*	0.292 (6)
C16B	0.692 (2)	0.6327 (14)	0.111 (4)	0.025 (7)*	0.292 (6)
H16B	0.6727	0.6739	0.0892	0.030*	0.292 (6)
C17B	0.601 (4)	0.594 (2)	0.131 (5)	0.041 (11)*	0.292 (6)
H17B	0.5281	0.6017	0.0944	0.049*	0.292 (6)
C18B	0.8654 (16)	0.6754 (9)	0.083 (3)	0.040 (4)*	0.292 (6)
H18D	0.8352	0.7023	−0.0077	0.061*	0.292 (6)
H18E	0.9353	0.6587	0.0418	0.061*	0.292 (6)
H18F	0.8772	0.6985	0.1908	0.061*	0.292 (6)
C19	0.8411 (3)	0.82014 (19)	0.6071 (6)	0.0318 (9)	
H19A	0.7774	0.8426	0.5776	0.038*	
C20	0.9073 (3)	0.79817 (19)	0.4741 (6)	0.0336 (9)	
H20A	0.8892	0.8048	0.3545	0.040*	
C21	1.0008 (3)	0.76615 (19)	0.5203 (6)	0.0336 (9)	
C22	1.0283 (3)	0.7563 (2)	0.6960 (6)	0.0342 (9)	
H22A	1.0928	0.7344	0.7254	0.041*	
C23	0.9611 (3)	0.77848 (16)	0.8261 (7)	0.0322 (8)	
H23A	0.9794	0.7723	0.9458	0.039*	
C24	0.8656 (3)	0.81016 (17)	0.7818 (5)	0.0265 (8)	

### Atomic displacement parameters (Å^2^)

**Table:**

	*U*^11^	*U*^22^	*U*^33^	*U*^12^	*U*^13^	*U*^23^
Br1	0.03289 (17)	0.0388 (2)	0.0375 (2)	−0.00330 (17)	0.0114 (2)	−0.0081 (3)
S1	0.0279 (4)	0.0316 (5)	0.0250 (4)	−0.0001 (4)	0.0058 (4)	0.0027 (4)
O1	0.0363 (16)	0.085 (3)	0.0320 (17)	0.0042 (17)	0.0021 (15)	−0.0159 (18)
O2	0.0474 (16)	0.0455 (18)	0.039 (2)	0.0191 (14)	0.0116 (15)	0.0103 (14)
O3	0.059 (2)	0.0385 (18)	0.058 (2)	−0.0128 (15)	0.0293 (18)	−0.0001 (16)
N1A	0.025 (3)	0.037 (4)	0.060 (6)	−0.002 (3)	0.007 (4)	0.006 (4)
C1A	0.068 (3)	0.033 (2)	0.096 (4)	0.0093 (18)	−0.032 (2)	−0.003 (2)
C2A	0.074 (6)	0.034 (4)	0.074 (7)	0.005 (3)	−0.048 (5)	−0.003 (4)
C3A	0.068 (3)	0.033 (2)	0.096 (4)	0.0093 (18)	−0.032 (2)	−0.003 (2)
C4A	0.068 (3)	0.033 (2)	0.096 (4)	0.0093 (18)	−0.032 (2)	−0.003 (2)
C5A	0.054 (6)	0.062 (6)	0.046 (6)	0.019 (5)	0.007 (5)	0.001 (5)
C6A	0.051 (7)	0.033 (5)	0.057 (6)	−0.002 (5)	−0.004 (6)	0.013 (3)
C7A	0.074 (6)	0.034 (5)	0.064 (6)	−0.018 (5)	−0.011 (5)	0.007 (4)
C8A	0.057 (4)	0.019 (3)	0.047 (4)	−0.014 (3)	0.001 (4)	−0.006 (3)
C9A	0.049 (4)	0.025 (4)	0.046 (4)	−0.014 (3)	−0.018 (4)	0.006 (3)
C10A	0.043 (4)	0.015 (3)	0.105 (7)	0.002 (3)	−0.046 (4)	0.001 (4)
C11A	0.068 (3)	0.033 (2)	0.096 (4)	0.0093 (18)	−0.032 (2)	−0.003 (2)
C12A	0.068 (3)	0.033 (2)	0.096 (4)	0.0093 (18)	−0.032 (2)	−0.003 (2)
C13A	0.026 (3)	0.040 (4)	0.040 (4)	0.010 (3)	−0.015 (3)	−0.016 (3)
C14A	0.029 (3)	0.028 (4)	0.034 (3)	−0.005 (3)	−0.006 (3)	−0.002 (3)
C15A	0.020 (3)	0.046 (5)	0.063 (5)	0.002 (3)	−0.001 (3)	0.015 (4)
C16A	0.018 (4)	0.038 (5)	0.040 (5)	0.008 (4)	−0.003 (4)	0.014 (3)
C17A	0.028 (4)	0.039 (5)	0.035 (6)	−0.001 (3)	0.006 (5)	−0.011 (5)
C18A	0.028 (3)	0.043 (4)	0.076 (6)	−0.007 (3)	0.007 (4)	0.002 (4)
C19	0.032 (2)	0.033 (2)	0.030 (2)	0.0022 (17)	0.0073 (18)	0.0056 (18)
C20	0.038 (2)	0.035 (2)	0.028 (2)	0.0010 (18)	0.0026 (19)	−0.0005 (17)
C21	0.0284 (18)	0.034 (2)	0.038 (2)	−0.0001 (18)	0.0107 (17)	−0.0008 (19)
C22	0.0301 (19)	0.038 (2)	0.034 (2)	0.0012 (18)	0.0032 (18)	0.0035 (18)
C23	0.0283 (15)	0.036 (2)	0.032 (2)	−0.0015 (14)	0.004 (2)	0.005 (2)
C24	0.0283 (16)	0.024 (2)	0.0275 (19)	−0.0038 (16)	0.0047 (15)	0.0039 (15)

### Geometric parameters (Å, °)

**Table:**

Br1—C21	1.905 (4)	C1B—C6B	1.37 (3)
S1—O2	1.433 (3)	C1B—C2B	1.45 (2)
S1—O1	1.439 (3)	C1B—C10B	1.50 (2)
S1—O3	1.453 (3)	C2B—C3B	1.41 (3)
S1—C24	1.780 (4)	C2B—H2BA	0.9500
N1A—C15A	1.357 (12)	C3B—C4B	1.47 (2)
N1A—C16A	1.410 (13)	C3B—H3BA	0.9500
N1A—C18A	1.485 (10)	C4B—C5B	1.42 (3)
C1A—C10A	1.407 (13)	C4B—H4BA	0.9500
C1A—C6A	1.428 (14)	C5B—C6B	1.33 (3)
C1A—C2A	1.465 (12)	C5B—H5BA	0.9500
C2A—C3A	1.361 (15)	C6B—C7B	1.36 (4)
C2A—H2AA	0.9500	C7B—C8B	1.42 (3)
C3A—C4A	1.374 (15)	C7B—H7BA	0.9500
C3A—H3AA	0.9500	C8B—C9B	1.43 (3)
C4A—C5A	1.368 (14)	C8B—H8BA	0.9500
C4A—H4AA	0.9500	C9B—C10B	1.30 (2)
C5A—C6A	1.448 (15)	C9B—H9BA	0.9500
C5A—H5AA	0.9500	C10B—C11B	1.57 (2)
C6A—C7A	1.453 (16)	C11B—C12B	1.552 (15)
C7A—C8A	1.398 (13)	C11B—H11B	0.9500
C7A—H7AA	0.9500	C12B—C13B	1.52 (2)
C8A—C9A	1.398 (9)	C12B—H12B	0.9500
C8A—H8AA	0.9500	C13B—C14B	1.35 (2)
C9A—C10A	1.369 (10)	C13B—C17B	1.52 (5)
C9A—H9AA	0.9500	C14B—C15B	1.32 (2)
C10A—C11A	1.454 (10)	C14B—H14B	0.9500
C11A—C12A	1.353 (10)	C15B—H15B	0.9500
C11A—H11A	0.9500	C16B—C17B	1.41 (5)
C12A—C13A	1.477 (10)	C16B—H16B	0.9500
C12A—H12A	0.9500	C17B—H17B	0.9500
C13A—C17A	1.382 (17)	C18B—H18D	0.9800
C13A—C14A	1.385 (9)	C18B—H18E	0.9800
C14A—C15A	1.350 (10)	C18B—H18F	0.9800
C14A—H14A	0.9500	C19—C24	1.383 (6)
C15A—H15A	0.9500	C19—C20	1.383 (6)
C16A—C17A	1.327 (18)	C19—H19A	0.9500
C16A—H16A	0.9500	C20—C21	1.388 (6)
C17A—H17A	0.9500	C20—H20A	0.9500
C18A—H18A	0.9800	C21—C22	1.398 (6)
C18A—H18B	0.9800	C22—C23	1.378 (6)
C18A—H18C	0.9800	C22—H22A	0.9500
N1B—C16B	1.17 (3)	C23—C24	1.399 (5)
N1B—C15B	1.41 (3)	C23—H23A	0.9500
N1B—C18B	1.52 (3)		
			
O2—S1—O1	114.1 (2)	C2B—C3B—C4B	123.4 (18)
O2—S1—O3	112.96 (19)	C2B—C3B—H3BA	118.3
O1—S1—O3	112.9 (2)	C4B—C3B—H3BA	118.3
O2—S1—C24	105.91 (18)	C5B—C4B—C3B	111.3 (17)
O1—S1—C24	105.27 (17)	C5B—C4B—H4BA	124.3
O3—S1—C24	104.71 (18)	C3B—C4B—H4BA	124.3
C15A—N1A—C16A	119.7 (8)	C6B—C5B—C4B	129 (2)
C15A—N1A—C18A	119.9 (7)	C6B—C5B—H5BA	115.7
C16A—N1A—C18A	120.4 (8)	C4B—C5B—H5BA	115.7
C10A—C1A—C6A	122.9 (9)	C5B—C6B—C7B	118 (2)
C10A—C1A—C2A	121.7 (9)	C5B—C6B—C1B	117 (3)
C6A—C1A—C2A	115.2 (10)	C7B—C6B—C1B	125 (2)
C3A—C2A—C1A	121.5 (10)	C6B—C7B—C8B	116 (2)
C3A—C2A—H2AA	119.2	C6B—C7B—H7BA	121.8
C1A—C2A—H2AA	119.2	C8B—C7B—H7BA	121.8
C2A—C3A—C4A	120.1 (10)	C7B—C8B—C9B	119.6 (18)
C2A—C3A—H3AA	119.9	C7B—C8B—H8BA	120.2
C4A—C3A—H3AA	119.9	C9B—C8B—H8BA	120.2
C5A—C4A—C3A	124.5 (12)	C10B—C9B—C8B	123.2 (17)
C5A—C4A—H4AA	117.8	C10B—C9B—H9BA	118.4
C3A—C4A—H4AA	117.8	C8B—C9B—H9BA	118.4
C4A—C5A—C6A	116.0 (11)	C9B—C10B—C1B	116.6 (16)
C4A—C5A—H5AA	122.0	C9B—C10B—C11B	124.9 (15)
C6A—C5A—H5AA	122.0	C1B—C10B—C11B	118.5 (12)
C1A—C6A—C5A	122.5 (10)	C12B—C11B—C10B	118.5 (11)
C1A—C6A—C7A	115.8 (10)	C12B—C11B—H11B	120.7
C5A—C6A—C7A	121.7 (10)	C10B—C11B—H11B	120.7
C8A—C7A—C6A	119.9 (9)	C13B—C12B—C11B	117.4 (13)
C8A—C7A—H7AA	120.0	C13B—C12B—H12B	121.3
C6A—C7A—H7AA	120.0	C11B—C12B—H12B	121.3
C7A—C8A—C9A	121.1 (7)	C14B—C13B—C17B	118 (2)
C7A—C8A—H8AA	119.4	C14B—C13B—C12B	116.9 (16)
C9A—C8A—H8AA	119.4	C17B—C13B—C12B	123 (2)
C10A—C9A—C8A	121.1 (8)	C15B—C14B—C13B	124.2 (18)
C10A—C9A—H9AA	119.4	C15B—C14B—H14B	117.9
C8A—C9A—H9AA	119.4	C13B—C14B—H14B	117.9
C9A—C10A—C1A	119.0 (7)	C14B—C15B—N1B	117.0 (17)
C9A—C10A—C11A	123.9 (8)	C14B—C15B—H15B	121.5
C1A—C10A—C11A	116.8 (7)	N1B—C15B—H15B	121.5
C12A—C11A—C10A	122.2 (7)	N1B—C16B—C17B	132 (3)
C12A—C11A—H11A	118.9	N1B—C16B—H16B	114.1
C10A—C11A—H11A	118.9	C17B—C16B—H16B	114.1
C11A—C12A—C13A	122.4 (7)	C16B—C17B—C13B	108 (3)
C11A—C12A—H12A	118.8	C16B—C17B—H17B	126.2
C13A—C12A—H12A	118.8	C13B—C17B—H17B	126.2
C17A—C13A—C14A	116.0 (8)	N1B—C18B—H18D	109.5
C17A—C13A—C12A	126.0 (8)	N1B—C18B—H18E	109.5
C14A—C13A—C12A	117.0 (6)	H18D—C18B—H18E	109.5
C15A—C14A—C13A	121.3 (6)	N1B—C18B—H18F	109.5
C15A—C14A—H14A	119.3	H18D—C18B—H18F	109.5
C13A—C14A—H14A	119.3	H18E—C18B—H18F	109.5
C14A—C15A—N1A	120.7 (6)	C24—C19—C20	121.6 (4)
C14A—C15A—H15A	119.6	C24—C19—H19A	119.2
N1A—C15A—H15A	119.6	C20—C19—H19A	119.2
C17A—C16A—N1A	117.4 (11)	C19—C20—C21	118.2 (4)
C17A—C16A—H16A	121.3	C19—C20—H20A	120.9
N1A—C16A—H16A	121.3	C21—C20—H20A	120.9
C16A—C17A—C13A	124.5 (13)	C20—C21—C22	121.3 (4)
C16A—C17A—H17A	117.7	C20—C21—Br1	119.0 (3)
C13A—C17A—H17A	117.7	C22—C21—Br1	119.7 (3)
C16B—N1B—C15B	119 (2)	C23—C22—C21	119.4 (4)
C16B—N1B—C18B	120 (2)	C23—C22—H22A	120.3
C15B—N1B—C18B	119.2 (18)	C21—C22—H22A	120.3
C6B—C1B—C2B	123.2 (18)	C22—C23—C24	120.0 (5)
C6B—C1B—C10B	117.5 (17)	C22—C23—H23A	120.0
C2B—C1B—C10B	118.7 (14)	C24—C23—H23A	120.0
C3B—C2B—C1B	115.8 (17)	C19—C24—C23	119.4 (4)
C3B—C2B—H2BA	122.1	C19—C24—S1	120.7 (3)
C1B—C2B—H2BA	122.1	C23—C24—S1	119.9 (3)
			
C10A—C1A—C2A—C3A	−173.3 (10)	C10B—C1B—C6B—C5B	168.5 (19)
C6A—C1A—C2A—C3A	2.1 (15)	C2B—C1B—C6B—C7B	179.5 (19)
C1A—C2A—C3A—C4A	−0.4 (16)	C10B—C1B—C6B—C7B	−10 (3)
C2A—C3A—C4A—C5A	−3.1 (17)	C5B—C6B—C7B—C8B	179 (2)
C3A—C4A—C5A—C6A	4.5 (17)	C1B—C6B—C7B—C8B	−2(4)
C10A—C1A—C6A—C5A	174.8 (10)	C6B—C7B—C8B—C9B	9(3)
C2A—C1A—C6A—C5A	−0.5 (16)	C7B—C8B—C9B—C10B	−2(3)
C10A—C1A—C6A—C7A	−3.7 (16)	C8B—C9B—C10B—C1B	−11 (2)
C2A—C1A—C6A—C7A	−179.0 (9)	C8B—C9B—C10B—C11B	165.7 (15)
C4A—C5A—C6A—C1A	−2.6 (17)	C6B—C1B—C10B—C9B	16 (2)
C4A—C5A—C6A—C7A	175.9 (10)	C2B—C1B—C10B—C9B	−172.8 (12)
C1A—C6A—C7A—C8A	4.2 (14)	C6B—C1B—C10B—C11B	−160.3 (17)
C5A—C6A—C7A—C8A	−174.4 (10)	C2B—C1B—C10B—C11B	10.7 (16)
C6A—C7A—C8A—C9A	−2.7 (13)	C9B—C10B—C11B—C12B	−6(2)
C7A—C8A—C9A—C10A	0.3 (11)	C1B—C10B—C11B—C12B	170.2 (13)
C8A—C9A—C10A—C1A	0.2 (12)	C10B—C11B—C12B—C13B	111.9 (15)
C8A—C9A—C10A—C11A	173.6 (8)	C11B—C12B—C13B—C14B	166.2 (15)
C6A—C1A—C10A—C9A	1.6 (14)	C11B—C12B—C13B—C17B	4(3)
C2A—C1A—C10A—C9A	176.6 (9)	C17B—C13B—C14B—C15B	7(3)
C6A—C1A—C10A—C11A	−172.3 (9)	C12B—C13B—C14B—C15B	−156.7 (17)
C2A—C1A—C10A—C11A	2.8 (13)	C13B—C14B—C15B—N1B	−7(3)
C9A—C10A—C11A—C12A	−26.5 (14)	C16B—N1B—C15B—C14B	12 (3)
C1A—C10A—C11A—C12A	147.0 (10)	C18B—N1B—C15B—C14B	175.3 (17)
C10A—C11A—C12A—C13A	173.6 (8)	C15B—N1B—C16B—C17B	−20 (5)
C11A—C12A—C13A—C17A	−9.3 (15)	C18B—N1B—C16B—C17B	177 (3)
C11A—C12A—C13A—C14A	158.9 (8)	N1B—C16B—C17B—C13B	18 (5)
C17A—C13A—C14A—C15A	−2.2 (10)	C14B—C13B—C17B—C16B	−9(3)
C12A—C13A—C14A—C15A	−171.6 (7)	C12B—C13B—C17B—C16B	153 (2)
C13A—C14A—C15A—N1A	0.8 (11)	C24—C19—C20—C21	−0.9 (6)
C16A—N1A—C15A—C14A	−2.1 (14)	C19—C20—C21—C22	−0.1 (6)
C18A—N1A—C15A—C14A	179.5 (7)	C19—C20—C21—Br1	−179.6 (3)
C15A—N1A—C16A—C17A	5.0 (16)	C20—C21—C22—C23	0.3 (7)
C18A—N1A—C16A—C17A	−176.6 (10)	Br1—C21—C22—C23	179.7 (3)
N1A—C16A—C17A—C13A	−7.0 (19)	C21—C22—C23—C24	0.6 (6)
C14A—C13A—C17A—C16A	5.6 (17)	C20—C19—C24—C23	1.7 (6)
C12A—C13A—C17A—C16A	173.9 (11)	C20—C19—C24—S1	−176.4 (3)
C6B—C1B—C2B—C3B	0.6 (16)	C22—C23—C24—C19	−1.6 (6)
C10B—C1B—C2B—C3B	−169.9 (11)	C22—C23—C24—S1	176.6 (3)
C1B—C2B—C3B—C4B	4.4 (14)	O2—S1—C24—C19	−23.2 (4)
C2B—C3B—C4B—C5B	−7(2)	O1—S1—C24—C19	−144.4 (3)
C3B—C4B—C5B—C6B	6(3)	O3—S1—C24—C19	96.4 (4)
C4B—C5B—C6B—C7B	177 (2)	O2—S1—C24—C23	158.7 (3)
C4B—C5B—C6B—C1B	−2(4)	O1—S1—C24—C23	37.5 (4)
C2B—C1B—C6B—C5B	−2(3)	O3—S1—C24—C23	−81.7 (3)

### Hydrogen-bond geometry (Å, °)

**Table:**

*D*—H···*A*	*D*—H	H···*A*	*D*···*A*	*D*—H···*A*
C2A—H2AA···O1^i^	0.95	2.53	3.392 (11)	151
C5A—H5AA···O2^ii^	0.95	2.47	3.362 (10)	157
C11A—H11A···O1^i^	0.95	2.34	3.282 (10)	175
C14A—H14A···O2^iii^	0.95	2.44	3.373 (7)	169
C16A—H16A···O3^iv^	0.95	2.49	3.361 (11)	153
C17A—H17A···O1^i^	0.95	2.35	3.227 (14)	153
C18A—H18A···O3^iv^	0.98	2.57	3.501 (8)	159
C19—H19A···O2	0.95	2.56	2.930 (5)	103
C20—H20A···O1^iv^	0.95	2.34	3.252 (5)	161
C22—H22A···O3^v^	0.95	2.51	3.274 (6)	137
C4A—H4AA···Cg2^vi^	0.95	2.84	3.659 (14)	145
C7A—H7AA···Cg4^vii^	0.95	2.88	3.657 (9)	140
C4B—H4BA···Cg2^vi^	0.95	2.90	3.59 (2)	130

Symmetry codes: (i) *x*−1/2, −*y*+3/2, *z*−1; (ii) −*x*+1/2, *y*−1/2, *z*−1/2; (iii) −*x*+3/2, *y*−1/2, *z*−1/2; (iv) *x*, *y*, *z*−1;  (v) *x*+1/2, −*y*+3/2, *z*; (vi) −*x*, −*y*+1, *z*+1/2; (vii) −*x*+1, −*y*+1, *z*−1/2.
